# Live demonstration versus procedural video: a comparison of two methods for teaching an orthodontic laboratory procedure

**DOI:** 10.1186/s12909-015-0479-y

**Published:** 2015-11-04

**Authors:** Nasser D. Alqahtani, Thikriat Al-Jewair, Khalid AL-Moammar, Sahar F. Albarakati, Eman A. ALkofide

**Affiliations:** 1 Department of Pediatric Dentistry and Orthodontics, College of Dentistry, King Saud University, P.O. Box 60169, Riyadh, Zip Code 11545 Saudi Arabia; 2 Department of Preventive Dental Sciences, College of Dentistry, University of Dammam, Dammam, Saudi Arabia

**Keywords:** Dental education, Dental undergraduates, Procedural video, Videotaped demonstrations, Orthodontics

## Abstract

**Background:**

To measure the effectiveness of procedural video compared to live demonstration in transferring skills for fabricating orthodontic Adam’s Clasp.

**Materials and Methods:**

Forty-nine fourth-year undergraduate male dental students were randomly assigned to two groups. The students in group A (*n* = 26) attended a live demonstration performed by one faculty, while students in group B (*n* = 23) watched a procedural video. Both the procedural video and live demonstration described identical steps involved in fabricating the Adam’s Clasp. Students in both groups were asked to fabricate an Adam’s Clasp in addition to completing a questionnaire, to measure their perceptions and satisfaction with the two teaching methods and lab exercise. Blind assessment was performed by one faculty for both groups.

**Results:**

The mean students’ scores in the fabrication of the Adam’s clasp were 6.69 and 6.78 for the live demonstration (group A) and the procedural video (group B), respectively. No significant difference was detected between the two groups (*P* = 0.864). Statistically significant difference was found in the mean response between the two groups for statement 6 on the questionnaire, “The steps in the teaching method were presented in a clear fashion and were easy to understand”. A higher mean response for group B was found compared to group A (*P* = 0.049). No significant differences were found between the two groups for the other statements (*P* > 0.05).

**Conclusion:**

Procedural video is equally as effective as a live demonstration. Both methods should be considered in teaching undergraduate orthodontic courses in order to improve the learning experience and to match different learning preferences of students.

## Background

Live demonstration to a group of students is a useful teaching tool in undergraduate orthodontic courses for dental students. Live demonstrations increase students’ confidence, improve communication skills, and provide better understanding of procedures than didactic teaching [1]. However, some factors have been shown to decrease their effectiveness such as shortage in faculty, small faculty to students’ ratio, difficulty in visualisation of the demonstration by students, and time constraints.

Recently, video demonstration or procedural video has been integrated into preclinical laboratory dental teaching [2, 3]. Procedural video can provide a valued teaching tool that allows better visualisation of laboratory steps, offer media-rich audio and visual stimulation covering a wider spectrum of the learning styles or preferences [4], allows students to review technical procedures before, during, or after the laboratory sessions, and overcomes shortage of faculty.

Several studies have investigated the efficacy of procedural video and live demonstration for teaching clinical and laboratory skills. Rosa et al. [5] found that visual methodologies is appropriate to develop and support students’ learning experiences. Mirkarimi et al. [6] found that both videotaped and live demonstrations were equally effective and can be used in combination or as an alternative for each other in teaching how to apply fissure sealant. In addition, Mir et al. [7] reported that videotaped demonstrations can be as effective as live demonstrations in transmitting clinical knowledge and skills to medical students. Packer et al. [2] tested the effectiveness of videotaped demonstrations as opposed to live demonstrations for the teaching of removable partial denture procedures. They concluded that both teaching methods developed a similar level of understanding of the principles behind the exercise, although students preferred the live demonstrations. Further, live demonstration enhances communication skills and increases student confidence [1]. However, teacher dependency of students, inadequate field of view, and non-repeatability of sessions are the main problems with this type of demonstration [8]. This study attempts to address the question of whether procedural video is more efficacious in the teaching of preclinical fabricate of orthodontic Adam’s Clasp on permanent molar tooth compared to traditional live demonstration, Hence the objective of this study was to measure the efficacy of procedural video compared to live demonstration in transferring skills to fourth-year undergraduate dental students during laboratory session and assess the students’ perceptions of the two methods used.

## Method

The current study was conducted at the College of Dentistry, King Saud University, Riyadh, Saudi Arabia. The study was reviewed ethically and approved by the Research Centre at the College of Dentistry (CDRC) at King Saud University (research project # FR 0148).

Forty-nine fourth-year male undergraduate dental students, who had not been exposed to any practical exercise on orthodontic wire bending, participated in this study. They were randomly assigned to two groups based on their serial number. The students in group A (*n* = 26) attended live demonstration performed by one faculty while students in group B (*n* = 23) watched a 12-minute procedural video on the projector. Both the live demonstration and the procedural video described identical steps involved in fabricating the Adam’s Clasp. Both groups were requested to fabricate an Adam’s Clasp on the maxillary permanent right first molar using duplicated orthodontic study models of the same patient. At the end of exercise, one blinded faculty member performed the assessment of the Adam’s Clasp for both groups according to pre-specified criteria (Table 1). The passing grade for this exercise was six out of ten which indicated that the student comprehended the minimum about the fabrication of the Adam’s clasp.

**Table 1:** Assessment criteria for the lab exercise

Criteria	Maximum possible mark
1. The bridge of the clasp should be straight	1
2. The bridge of the clasp shouldn’t touch the buccal surface of the 1st molar	1
3. The right loop should touch the buccal surface at the corner,	1
4. The left loop should touch the buccal surface at the corner,	1
5. The right arm of the clasp should follow the occlusal embrasure,	1
6. The left arm of the clasp should follow the occlusal embrasure,	1
7. The right arm of the clasp should touch the occlusal embrasure.	1
8. The left arm of the clasp should touch the occlusal embrasure.	1
9. When the right arm goes on the palatal tissue, there should be about 0.5 to 1 mm clearance.	1
10. When the left arm goes on the palatal tissue, there should be about 0.5 to 1 mm clearance.	1
Total	10

An 8-item questionnaire was developed for the purpose of this study in order to measure the students ‘perception towards the two teaching methods and lab exercise by using a Likert scale with five response options (1 = strongly disagree, 2 = disagree, 3 = uncertain, 4 = agree, and 5 = strongly agree). The questionnaire was pre-tested on 15 fifth year students who were not part of this study and the questions were modified accordingly. Statements in the questionnaire were designed to measure the students ‘level of stress during the exercise, their ability to fabricate Adam’s Clasp exercise, and their satisfaction with the teaching method that they have received. The students completed the survey at the end of the exercise and the results were collected anonymously on SurveyMonkey (www.surveymonkey.com).

Statistical analysis was conducted using the Statistical Package for Social Sciences (Version 16.0; SPSS Inc., Chicago, IL, USA). Mean differences between the groups in the lab exercise scores were assessed using independent samples t-test. An alpha of 0.05 was used as the level of significance.

## Results

Tables 2, 3 shows the mean scores obtained by each group in the lab exercise. The mean students’ scores for live demonstration (group A) and procedural video (group B) were 6.6 and 6.7, respectively. No significant difference was detected between the two groups (*P* > 0.05).

**Table 2:** Mean scores obtained by students of both groups in the blind assessment of the lab exercise

	Live	Video
N		
Mean(SD)		
Mean Diff		
P-value		
95 % CI

**Table 3:** Mean scores obtained by students of both groups in the blind assessment of the lab exercise

Study groups	N	Mean	SD	P-value
Live Demonstration	26	6.69	1.4	.864*
Procedural Video	23	6.78	2.1	
Total	49	6.73	1.8	

*No significant difference at *P* < 0.05 (Independent sample t-test)

Among the 49 students, 42 students (22 students from group A and 20 students from group B) completed the questionnaire. This represents a response rate of 85.7 %. Table 4 shows a comparison between groups’ responses to the questionnaire. Independent samples t-test indicated statistically significant differences in the mean response between the two groups for statement 6 on the questionnaire, “The steps in the teaching method were presented in a clear fashion and were easy to understand”. A higher mean response for group B was found compared to group A (*P* = 0.049). No significant differences were found between the two groups for the other statements (*P* > 0.05).

**Table 4:** Students’ perception of the live demonstration and procedural video methods

Question	Study groups	N	Mean	SD	P-value
I felt stressful during wire bending exercise.	Live Demonstration	22	3.09	1.192	.639
	Procedural Video	20	3.25	.967	
It was easy to perform wire bending exercise.	Live Demonstration	22	3.05	1.065	.662
	Procedural Video	20	2.95	.999	
I was satisfied with my performance in wire bending exercise.	Live Demonstration	22	3.05	1.133	.535
	Procedural Video	20	3.25	.967	
The teaching method was helpful to perform wire bending exercise.	Live Demonstration	22	3.55	.912	.723
	Procedural Video	20	3.65	.988	
The teaching method was adequate for performing wire bending exercise.	Live Demonstration	22	3.64	.848	.397
	Procedural Video	20	3.40	.940	
The steps in teaching method presented in clear fashion and easy to understand.	Live Demonstration	22	3.18	1.220	.049**
	Procedural Video	20	3.80	.696	
I prefer the teaching method that I have received over the other teaching method.	Live Demonstration	22	3.59	1.008	.142
	Procedural Video	20	3.10	1.119	
The teaching method needs further improvement to support my learning	Live Demonstration	22	3.41	1.054	.190
	Procedural Video	20	3.85	1.089	

**Significant difference at *P* < 0.05

## Discussion

New technology has broadened the teaching methods available for learners. Several studies [5–7] have tested the procedural video approach to educate dental students and has been shown to be as effective as live demonstration. Therefore, the current study was undertaken to assess the procedural video demonstration in comparison to the live demonstration to overcome some of the common problems faced during traditional teaching methods which include but are not limited to; short laboratory time, and shortage of teaching faculty which makes ideal student to instructor ratio not possible.

The evaluation of students’ performance on fabrication of Adam’s clasp in both groups was done by one assessor because in every lab exercise the students are evaluated by one faculty member for fair distribution of the grades and consistent evaluation. The evaluation of both groups showed no significant difference, which indicates a similar level of understanding gained by both groups. This finding is similar to what Packer et al. [2] found when comparing between videotaped and live demonstration for teaching removable partial denture procedures. In the current study, the mean students’ scores for live demonstration (group A) and procedural video (group B) were 6.69 and 6.78, respectively. These low scores may be due to the lack of previous exposure to any practical exercise on Adam’s clasp fabrication. The results also showed that there was no significant difference in the level of stress between the two groups. This finding is in line with those obtained by Rosa et al. [5], Mirkarimi et al. [6] and Mir et al. [7].

Approximately 59 % from the live demonstration group and 40 % from the procedural video group preferred the teaching method they received. However, 35 % of the students from the procedural video group did not prefer their teaching method compared to only 18.18 % from the live demonstration group. This preference for live demonstration has been observed in similar studies by Bazyk et al [9] and Packer et al. [1]. The students in Bazyk et al. study [9] believed that the live demonstration gave them the opportunity to ask questions and interact with the instructor. Further, Packer et al. [1] found that live demonstration increases student confidence, improves communication skills, and provides better understanding of procedures. On the other hand, the results in the current study revealed a significant difference in mean response between the two groups for statement 6 on the questionnaire, which measures the clearness and ease of understanding for the steps in the teaching methods. A higher mean response for procedural video group was found compared to live demonstration group (*P* = 0.049). The procedural video provides better visualisation of laboratory steps and allows students to review technical procedures before, during, or after the laboratory sessions. This is in line with Aragon and Zibrowski study [10] in which students in the clinic, who were given a copy of a video, reported that they can see better on video versus a demonstration with everyone crowding around, and that the procedure can be reviewed at any time.

One limitation of this study is the lack of female participants. Female students go to a different female campus and are being taught by different faculty, which made their enrolment logistically difficult.

The students in this study were reluctant to replace live demonstration with procedural videos. This might be because they are accustomed to attending traditional live demonstration. Sharing the results of the studies that compare the effectiveness and reliability between videotaped and live demonstration with students may reassure them and gain their confidence about procedural videos.

## Conclusion

Carefully designed and developed procedural video is equally as effective as a live demonstration. Each method has its own advantages and limitations, therefore both methods should be considered in teaching undergraduate orthodontic courses in order to improve learning experiences and to match different learning preferences of students.
